# Variations in centre of pressure and balance performance induced by footwear drop in healthy adults

**DOI:** 10.1007/s00264-025-06664-4

**Published:** 2025-10-01

**Authors:** Raquel Fragua-Blanca, Natalia Tovaruela-Carrión, Manuel Jesús Tena-León, Elena Escamilla-Martínez

**Affiliations:** 1 Departamento de Enfermería, Fisioterapia y Terapia Ocupacional. Facultad de Ciencias de la Salud, Universidad de Castilla-La Mancha, 45600 Talavera de la Reina,, Spain; 2 Departamento de Podología. Facultad de Enfermería, Fisioterapia y Podología, Universidad de Sevilla, 41009 Sevilla,, Spain; 3 Investigador independiente, Práctica privada, 07010 Palma de Mallorca,, Spain; 4 Departamento de Enfermería. Centro Universitario de Plasencia, Universidad de Extremadura, 10600 Plasencia,, Spain

**Keywords:** Drop, Sports footwear, Stabilometry, Centre of pressure, Dinascan/IBV^®^, Foot

## Abstract

**Background:**

Posturography is a diagnostic technique that quantifies postural control through Centre of Pressure (CoP) displacement analysis on a force platform. Footwear characteristics, particularly heel-to-toe drop, may influence balance by modifying plantar pressure distribution and proprioceptive feedback. The aim of this study was to evaluate the impact of different footwear drops (0 mm, 5 mm, 10 mm) on postural control in healthy young adults, considering sex, BMI, and shoe size.

**Methods:**

A cross-sectional study was conducted in 117 participants (56 men, 61 women) using the Dinascan/IBV^®^ platform and the Romberg test. CoP displacement and velocity were analyzed.

**Results:**

Significant differences were observed in CoP total displacement (*p* < 0.001), mean velocity (*p* < 0.001), and medio-lateral dispersion (*p* = 0.024) when comparing 0 mm to 5 mm and 10 mm drops. Sex differences were significant at 0 mm drop for maximum medio-lateral force (*p* < 0.001) and mean velocity (*p* = 0.042), with men exhibiting greater values. At 5 mm drop, men showed significantly higher swept area (*p* = 0.029) and anteroposterior displacement (*p* = 0.007) than women.

**Conclusions:**

Small variations in footwear drop can affect postural control, particularly in the medio-lateral plane. Sex and BMI significantly influence CoP behavior, suggesting the need to consider these factors in footwear design and clinical balance assessments.

## Introduction

Posturography is a tool used to assess postural control and was recognized as a diagnostic and therapeutic method in 1985 by the French Association of Posturology [1]. This technique quantifies the oscillations of the Centre of Pressure (CoP) in the sagittal and coronal planes, recording body sway using a platform equipped with force transducers. These devices measure body movements, and the collected data is analyzed using specialized software [2, 3].

Postural control depends on the central nervous system’s ability to integrate and process visual, vestibular, and proprioceptive stimuli [4, 5]. Factors such as base of support, foot positioning, and the condition of the musculoskeletal system also significantly influence balance and contribute to maintaining postural stability [4].

Posturography allows for the identification of dysfunction severity, compensation levels, and the individual’s adaptive capacity. This enables the customization of treatment based on each person’s specific needs [6].

From a postural perspective, the causes of imbalance are diverse and may include altered plantar sensory input, oculomotor conditions, and musculoskeletal disorders [1, 7].

To ensure accurate posturographic testing, it is essential to eliminate external visual and auditory stimuli, creating an environment without light or noise to allow for reliable measurements [8].

During evaluation, the individual’s postural movements are assessed by analyzing the CoP displacement over a set period. The recorded CoP signal can be represented in two ways: as a stabilogram, which reflects CoP movements in the anteroposterior (AP) and mediolateral (ML) directions, or as a statokinesigram, which provides a vectorial representation of the body’s center of gravity (CG) projection [9, 10].

The Romberg test is considered the gold standard for assessing postural control in posturography [11]. First described by Moritz Heinrich Romberg, this test evaluates the integrity of the proprioceptive pathway and is considered positive when the individual, upon closing their eyes, begins to sway laterally and loses balance, potentially falling without support.

The gold standard test for postural control in posturography is the Romberg test. This test was described by Moritz Heinrich Romberg and assesses the integrity of the proprioceptive pathway [12, 13]. A Romberg test is considered positive when the individual, upon closing their eyes, begins to sway immediately from side to side, losing balance and possibly falling if not supported [13].

In this context, and considering the importance of understanding how factors such as drop, sex, shoe size, and the Body Mass Index (BMI) can influence postural balance, the present study aims to: quantify the changes in the CoP with the three drops studied, analyze CoP differences based on sex, determine CoP differences according to shoe size, and observe the changes in CoP according to BMI. These objectives will provide a more detailed and accurate understanding of the variables that affect postural control during walking, thus contributing to a greater understanding of the underlying mechanisms in postural stability.

## Materials and methods

### Designing

An observational, descriptive, and cross-sectional study was conducted in compliance with the ethical principles outlined in the Declaration of Helsinki and the Biomedical Research Act 14/2007. The study design was approved by the University of XXX Research Ethics Committee (ID: 137//2023). All participants signed informed consent forms to participate in the study.

The study was conducted and reported following the Strengthening the Reporting of Observational Studies in Epidemiology (STROBE) guidelines.

### Sample

The sample consisted of 117 participants (56 males and 61 females), students of the Podiatry degree program at the University xxx, Center xxx. Subjects were between 18 and 28 years old, with a mean age of 21.63 ± 2.4 years, a mean weight of 71.08 ± 14.05 kg, a mean height of 169.29 ± 9.3 cm, and a mean BMI of 25.02 ± 5.59 kg/m². Inclusion criteria were: (a) Age between 18 and 30 years, (b) Physical and mental capability to voluntarily participate by signing informed consent, (c) Absence of any pathology or surgery affecting normal balance. Exclusion criteria involved not meeting these requirements.

### Data collection protocol

**Fig. 1:**
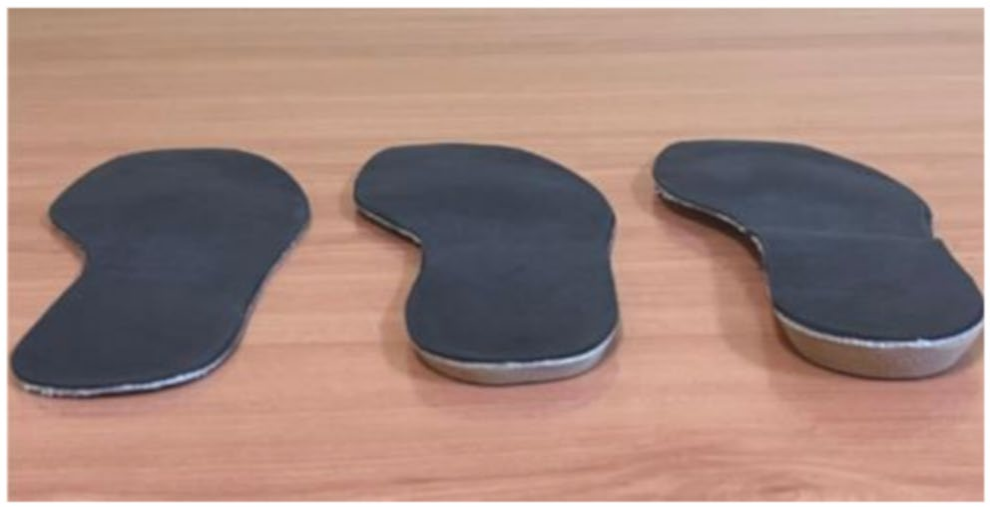
Insoles used for data collection, from left to right: 0 mm, 5 mm, and 10 mm drop

**Fig. 2:**
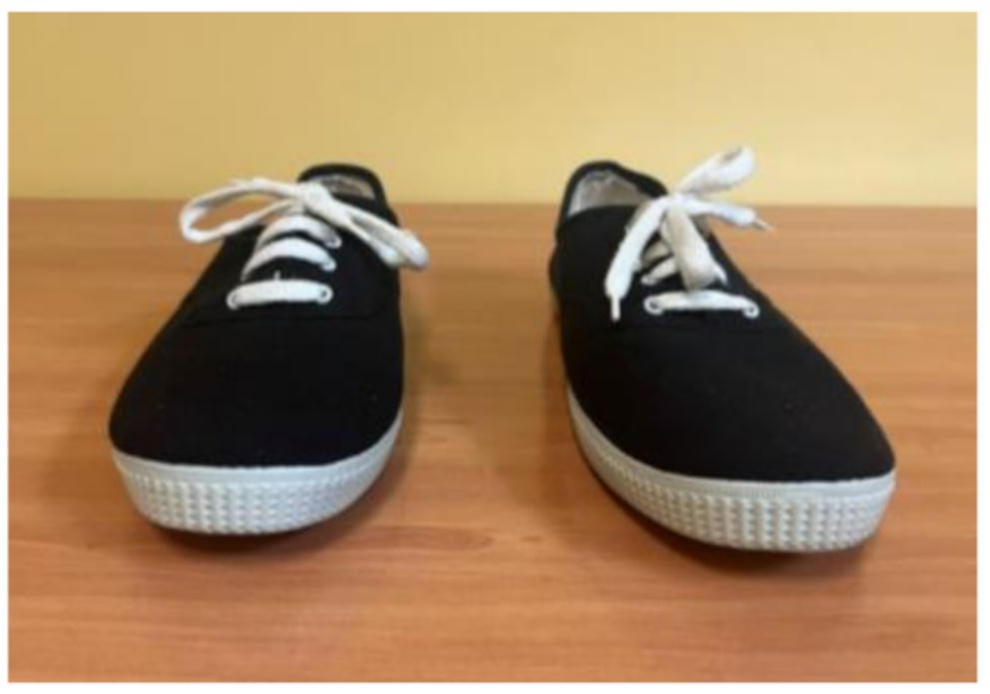
Shoes used for data collection

For data collection, custom insoles with different heel-to-toe drops were specifically manufactured for this study. Each insole consisted of a 1.9 mm rigid resin base, a high-density EVA layer (Shore A hardness 70º) to simulate the 5 mm and 10 mm drops, and a 1 mm micro-perforated top cover providing comfort and breathability (Fig. 1). EVA was selected for its mechanical properties, particularly its resistance to deformation after repeated use. The insoles were designed to fit securely inside the shoe, ensuring stable placement during stabilometric assessments. The insoles were placed in standard lace-up canvas shoes (Fig. 2).

The footwear model was selected due to its neutral characteristics, featuring no initial heel-to-toe drop and a highly simplified structure without motion control elements, arch supports, or rigid reinforcements. This choice allowed us to establish standardized baseline conditions and reduce potential external influences on the stabilometric assessments performed in this study, prior to introducing the insoles with different drop heights.

**Fig. 3:**
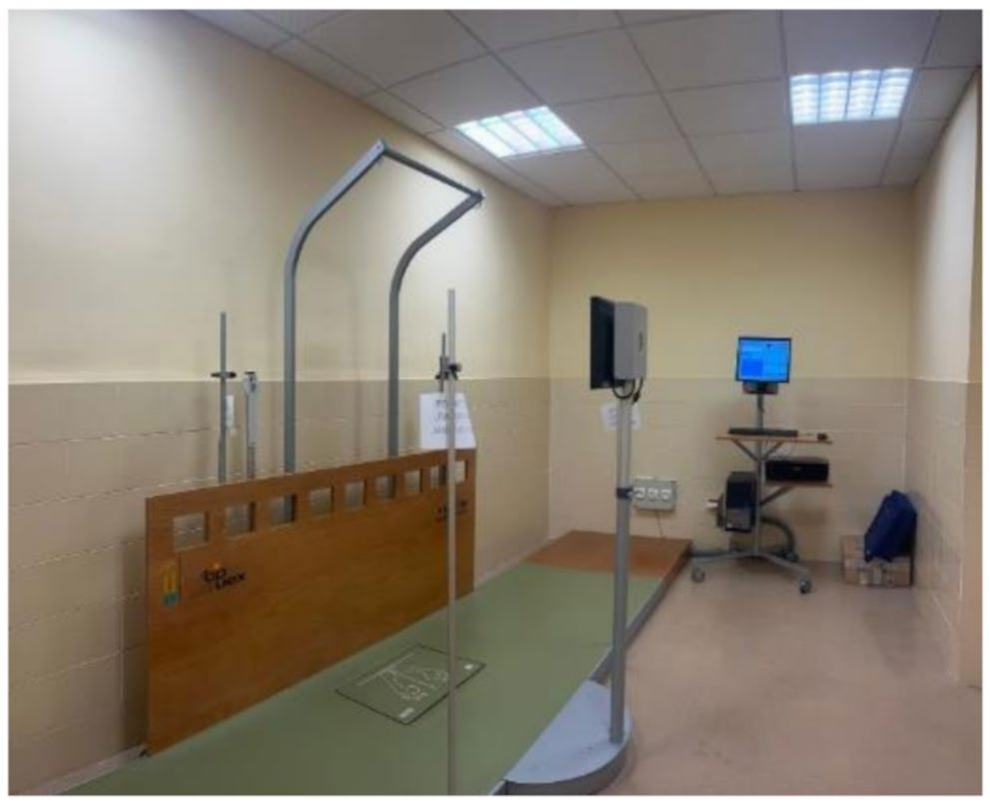
Dinascan/IBV^®^ force platform

The stabilometry study was evaluated using the Dinascan/IBV^®^ platform (600 × 370 mm active area, 100 mm height, and 25 kg), integrated with the NedSVE/IBV^®^ system (Valencia, Spain) (Fig. 3).

Before measurements, the platform was calibrated, and the subject’s weight was recorded by standing on it.

**Fig. 4:**
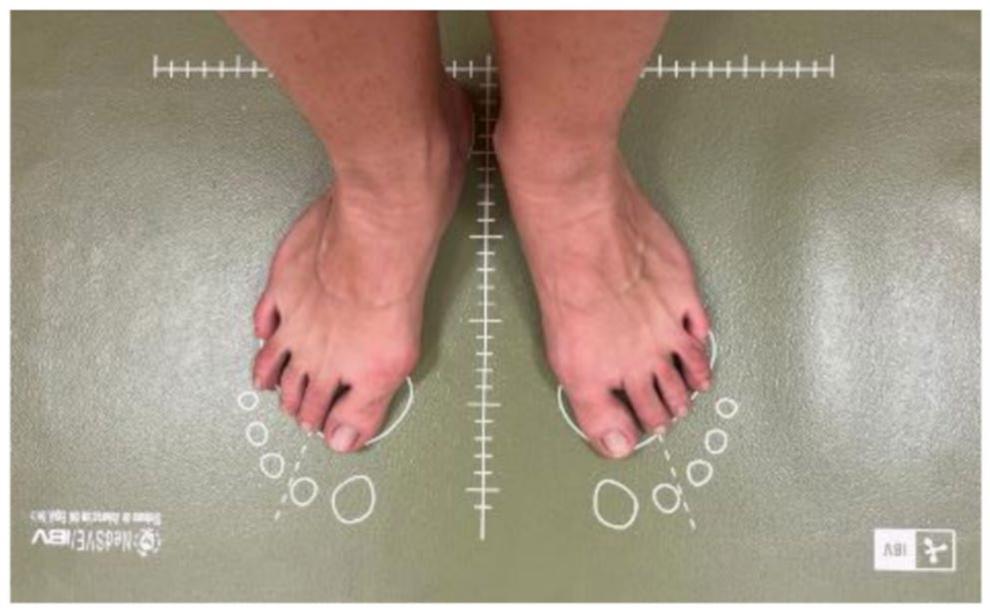
Foot placement for the Romberg test

The participant was then instructed to adopt a vertical, upright, and comfortable posture, with arms relaxed at the sides of the body and feet slightly angled outward (Fig. 4).

The balance evaluation was carried out following this protocol:

First, the subject was asked to step onto the platform for weighing and position their feet according to the marked footprints. The feet were to form a 30º angle, keeping the heels together without the internal malleoli touching, ensuring that the second metatarsal was aligned with the discontinuous line marked on the platform, known as the Vertical Barré.

Next, the participant was asked to fix their gaze on a stationary point at eye level and remain still, silent, and motionless for a period of 30 s. This interval corresponds to the duration of the Romberg test with eyes open (ROA).

To ensure accurate measurements and avoid external distractions, the room was maintained in complete silence during the measurements.

**Fig. 5:**
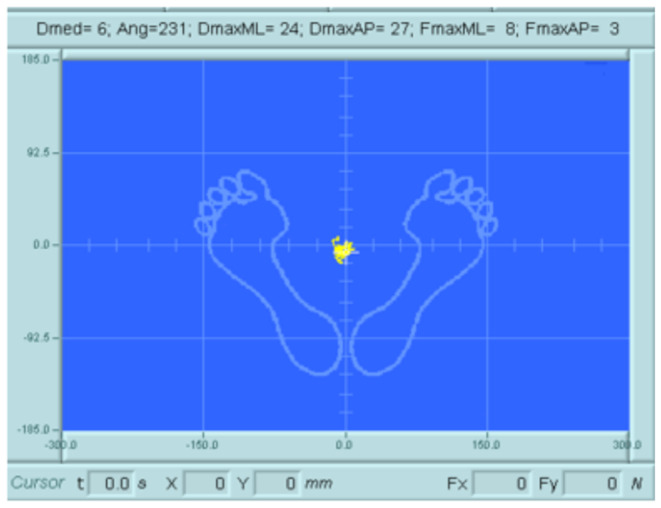
Representation of the Centre of Gravity Using a Statokinesiogram

The movements and postural changes of the individual are obtained through the analysis of the CoP trajectory over a given period of time [8]. The recorded CoP signal can be visualized in two ways: through an estabilogram, which represents the CoP movements in the AP or ML direction, and a statokinesiogram (Fig. 5), which is a vectorial representation of the projection of the centre of gravity (CG) [10, 14].

### Statistical analysis

Statistical analysis was conducted using IBM SPSS Statistics 27.

The variables studied were *ROA total displacement*, *ROA displacement angle*, *ROA ML dispersion*, ROA *AP dispersion*, *ROA swept area*, *ROA mean velocity*, *ROA ML displacement*, *ROA AP displacement*, *ROA ML maximum force*, *and ROA AP maximum force.*

Inferential analysis was conducted to draw conclusions following the formulation of statistical hypotheses regarding the study variables. Initially, a descriptive statistical analysis was performed for the balance analysis, where the mean and standard deviation (SD) of all variables assessed in the ROA test were calculated. Subsequently, repeated measures ANOVA and the Friedman test for related samples were applied to perform a two-factor variance analysis by ranks. A post hoc test (pairwise comparison) was then conducted to identify which specific drop resulted in the greatest variations. For comparisons of continuous variables that adhered to a normal distribution, the Independent Samples T-Test was employed. In cases where the assumptions of normality were not met or where the variables were ordinal in nature, the Mann-Whitney U Test for independent samples was applied. The significance level was set at *p* < 0.05.

## Results

First, descriptive analysis of the sample was conducted to obtain mean and standard deviation (SD) values (Table 1).

**Table 1:** Sample descriptive statistical analysis

Variables	0 mm		5 mm		10 mm	
	Mean	SD	Mean	SD	Mean	SD
**ROA total displacement** (*mm*)	8.8	9.6	12.0	7.3	11.7	6.7
**ROA displacement angle** (*º*)	182.2	96.5	**193.1**	100.0	168.3	101.8
**ROA ML dispersion** (*mm*)	2.6	1.0	2.9	1.1	2.9	1.1
**ROA AP dispersion** (*mm*)	3.9	1.5	4.2	2.2	3.9	1.6
**ROA swept area** (*mm²*)	38.9	24.0	47.5	41.1	45.3	33.7
**ROA mean velocity** (*m/s*)	**0.011**	0.002	**0.011**	0.002	**0.011**	0.002
**ROA ML displacement** (*mm*)	13.2	4.6	14.3	5.2	15.3	6.1
**ROA AP displacement** (*mm*)	18.6	6.0	19.9	8.9	19.0	7.1
**ROA maximum ML force** (*N*)	6.9	2.8	7.0	2.9	7.2	3.1
**ROA maximum AP force** (*N*)	3.9	1.5	4.3	1.8	4.5	2.3

Subsequently, the ANOVA and Friedman tests for related samples. The results revealed statistically significant differences in *ROA displacement*, *ROA ML dispersion*, *ROA mean velocity*, *ROA ML displacement*, *and ROA maximum AP force*, with *p* values < 0.05 (Table 2).

**Table 2:** Significance (*p*-value) of the different studied variables

Variables	*p*
**ROA total displacement** (*mm*)	< 0.001
**ROA ML dispersion** (*mm*)	0.024
**ROA mean velocity** (*m/s*)	< 0.001
**ROA ML displacement** (*mm*)	0.004
**ROA maximum AP force** (*N*)	0.048

Finally, a post hoc test (pairwise test) was conducted (Table 3) to determine with which drop the greatest variations were observed.

**Table 3:** Post hoc pairwise test for the variables that showed significant differences in the previous analyses

Variables		0–5 mm	0–10 mm	5–10 mm
**ROA total displacement** (*mm*)		**< 0.001**	**< 0.001**	> 0.999
**ROA ML dispersion** (*mm*)		**0.032**	0.118	> 0.999
**ROA mean velocity** (*m/s*)		**0.007**	**0**	0.509
**ROA ML displacement** (*mm*)		0.150	**0.004**	0.607
**ROA maximum AP force** (*N*)		0.307	0.051	> 0.999

Regarding total displacement and mean velocity in the ROA condition, statistically significant differences were observed when comparing drop 0 with drop 5, as well as drop 0 with drop 10.

As for *ROA ML dispersion*, significant differences were only found between drop 0 and drop 5. In terms of *ROA ML displacement*, statistically significant differences were identified when comparing drop 0 with drop 10. It is important to note that although *ROA maximum AP force* showed a statistically significant result (*p* = 0.048), the post hoc analysis revealed no significant differences between any of the evaluated conditions.

Additionally, potential differences in stabilometric behavior between women and men in response to the different drop levels proposed under the ROA condition were examined (Table 4). Depending on the distribution of the data, either the Independent Samples T-Test (for normally distributed variables) or the Mann-Whitney U Test (for non-normally distributed or ordinal variables) was applied to compare the two groups.

**Table 4:** Influence of sex on stabilometric behavior during the ROA condition across the different proposed drop levels

Variables	Drop	Men		Women		*p*
		Mean	SD	Mean	SD	
**ROA total displacement** (*mm*)	**0 mm**	11.0 12.0 11.4	12.2 7.9 6.3	6.8 11.9 12.1	5.9 6.8 7.0	0.083
	**5 mm**					0.849
	**10 mm**					0.612
**ROA displacement angle** (*º*)	**0 mm**	197.1 197.4 159.0	97.2 98.8 97.4	168.6 189.1 176.8	94.6 101.7 105.7	0.078
	**5 mm**					0.623
	**10 mm**					0.331
**ROA ML dispersion** (*mm*)	**0 mm**	2.8 3.1 3.0	1.1 1.4 1.1	2.5 2.7 2.8	1.0 0.9 1.0	0.287
	**5 mm**					0.230
	**10 mm**					0.129
**ROA AP dispersion** (*mm*)	**0 mm**	4.3 4.7 4.0	1.7 2.5 1.6	3.6 3.7 3.9	1.2 1.7 1.6	**0.045**
	**5 mm**					0.020
	**10 mm**					0.889
**ROA swept area** (*mm²*)	**0 mm**	43.1 57.5 48.5	26.9 52.1 39.1	35.1 38.3 42.3	20.4 24.5 28.0	0.101
	**5 mm**					**0.029**
	**10 mm**					0.438
**ROA mean velocity** (*m/s*)	**0 mm**	0.011 0.012 0.012	0.002 0.002 0.002	0.010 0.011 0.011	0.002 0.002 0.002	**0.042**
	**5 mm**					0.007
	**10 mm**					0.038
**ROA ML displacement** (*mm*)	**0 mm**	13.9 15.4 16.2	4.5 5.9 6.7	12.7 13.3 14.5	4.6 4.3 5.4	0.180
	**5 mm**					0.050
	**10 mm**					0.183
**ROA AP displacement** (*mm*)	**0 mm**	19.6 21.6 19.8	6.9 8.9 8.1	17.7 18.4 18.3	4.8 8.8 6.0	0.152
	**5 mm**					**0.007**
	**10 mm**					0.543
**ROA maximum ML force** (*N*)	**0 mm**	7.8 8.0 7.8	3.0 3.0 2.9	6.1 6.2 6.6	2.3 2.5 3.2	**< 0.001**
	**5 mm**					< 0.001
	**10 mm**					0.006
**ROA maximum AP force** (*N*)	**0 mm**	4.5	1.5	3.4 3.8 3.9	1.4 1.6 1.7	**< 0.001**
	**5 mm**	4.8	1.7			< 0.001
	**10 mm**	5.2	2.7			0.001

Statistically significant differences were found in *ROA anteroposterior dispersion*, *ROA mean velocity*, *ROA maximum mediolateral force*, and *ROA maximum AP force* under the 0 mm drop condition. This suggests that any subsequent differences observed could be attributed to the sex of the participants (male or female), rather than to a differential response to the increase in drop to 5–10 mm.

In contrast, for *ROA swept area* and *ROA AP displacement*, no significant differences were observed between men and women under the 0 mm drop condition. However, when analyzing the 5 mm drop, both parameters were significantly higher in men compared to women, with *p*-values of 0.029 and 0.007, respectively.

## Discussion

Stabilometric measurements were performed using the Dinascan/IBV^®^ force platform (Instituto de Biomecánica de Valencia, Spain), which has been previously employed in postural control research showing good reliability [15]. The experimental protocol was based on the standardized Romberg test, in the eyes-open condition, with subjects standing upright, heels together, feet externally rotated at approximately 30°, and arms relaxed alongside the body. The instrumented Romberg test has been validated as a reliable method for postural stability and fall-risk screening [16, 17], situating the present work within widely recognized procedures in stabilometry.

The results show statistically significant differences in the variables CoP displacement and CoP mean velocity when comparing the 0 mm drop condition with both 5 mm and 10 mm drops. However, for the variable CoP ML dispersion, significant differences were only observed between the 0 mm and 5 mm drop conditions. This behavioural difference may be explained by considering the role of the multiple factors involved in balance control, including mechanical, physiological, psychological, environmental, and individual physical condition-related factors [18, 19].

As the drop increased progressively, it is possible that the somatosensory system, through mechanoreceptors, responded to compensate for the initial imbalance by promoting postural adaptation. This adaptation could explain the absence of significant differences in some comparisons, such as between the 5 mm and 10 mm drop conditions. Furthermore, the fact that participants were young and free of balance-impairing pathologies may have influenced their ability to employ effective compensatory mechanisms, similar to what is observed in patients with vestibular dysfunction who rely on visual information to maintain stability [20].

With regard to postural strategies, it is well established that the body initially relies on the ankle strategy to control anteroposterior displacements. When instability increases, the hip strategy is activated, which is associated with mediolateral movements. In more extreme situations, the stepping strategy is employed [21]. It is possible that the increase in drop from 0 to 5 mm generated a sensation of instability comparable to that described by Peydro de Moya et al. [22] when analyzing unstable surfaces, thus requiring a postural response from the hip.

Regarding the ML displacement of the CoP, significant differences were found only between 0 mm and 10 mm of drop. This finding suggests that a higher heel elevation increases ML instability in a static position, which is relevant, as this type of instability has been associated with a higher risk of falls [23]. Additionally, ML imbalance is also related to falls during walking [24]. Therefore, even small heel elevations could have a negative impact on balance in young and healthy individuals, leading to the idea that these effects could be amplified in older populations or those with pathologies that compromise postural control. Finally, it is important to remember that the human body is naturally in a slight anterior imbalance. The line of gravity is located in front of the malleoli, and approximately two-thirds of the head’s weight falls in front of this line. This configuration requires constant tension in the posterior muscle chain, which could explain why the antero-posterior displacement of the CoP was smaller than the medio-lateral displacement [25].

When analyzing the differences in CoP based on sex, significant differences were observed in the variables ROA AP dispersion, ROA mean velocity, ROA maximum ML force, and ROA maximum AP force, starting from the initial condition with 0 mm drop. As previously noted in the gait analysis, these differences may be associated with the anatomical particularities between men and women [26–29], which makes it less appropriate to continue the analysis with the 5 mm and 10 mm drops in these variables. In this context, if new differences are detected, it is likely that they would be mainly due to the participants’ sex and not the drop effect itself.

On the other hand, the variables ROA swept area, ROA ML displacement, and ROA AP displacement did not show significant differences between sexes in the 0 mm drop condition, which justifies their comparative analysis at the 5 mm and 10 mm drop levels. When analyzing the results with a 5 mm drop, higher values were observed in men compared to women in all these variables, with statistically significant differences (*p* = 0.029, *p* = 0.050, and *p* = 0.007, respectively). A possible explanation for these results lies in the differences in muscular, fascial, and ligamentous elasticity between men and women, influenced by hormonal factors. The scientific literature has highlighted how hormones play a key role in the flexibility of female fasciae [30–32]. These hormones allow female connective tissue to adapt to the various structural changes that occur throughout life. In this regard, it has been observed that variations in hormonal levels can modify the ratio of type I and type III collagen, as well as other structural proteins like fibrillin and elastin, leading to significant changes in the behaviour of the musculoskeletal tissue [30].

Regarding the influence of height on the CoP oscillations, the results show some association, although with low correlation coefficients in all the variables analyzed, none exceeding a value of 0.5. The highest coefficient was 0.435, corresponding to the ROA maximum ML force with 5 mm of drop. This relationship can be explained by the position of the centre of gravity in relation to height, as according to Plas et al. [33], this centre is located approximately at 55% of the subject’s height in a relaxed bipedal position, meaning that with greater height, the centre of gravity is higher.

It is worth noting that the sample in this study was quite homogeneous in terms of height, with a mean of 169.29 cm, which likely contributed to the low strength of the correlations found. Similarly, age is also a widely recognized factor in the literature for its impact on postural control.

Classic research, such as that by Sheldon [34], already identified that optimal postural control is reached in late adolescence and remains stable until around 60 years of age, after which it begins to deteriorate more rapidly.

In this regard, Era et al. [35] also observed balance impairments at relatively early ages, noting a greater decline starting from the sixth decade of life.

This deterioration in balance during adulthood has been associated with multiple factors, such as the loss of muscle mass and strength, increased body weight, fatigue, reduced ligament flexibility, and a general decline in reaction time [36]. Furthermore, several studies have recorded greater displacements and velocities of the CoP in older adults compared to younger individuals, reflecting an increase in the activation of automatic postural responses and the use of compensatory strategies, such as the ankle or hip strategy [37, 38].

For all these reasons, it is reasonable to think that the low correlation observed in our study between height and CoP may be due, in addition to the homogeneity in stature, to the relatively young and uniform age of the sample.

The results obtained when relating the CoP oscillations to the BMI show a direct relationship between BMI and CoP oscillations. In the variables of ROA maximum force mediolateral and anteroposterior, a positive correlation was observed between BMI and oscillations. The differences in BMI were recorded in the initial condition with 0 drop, making it difficult to determine whether the variations observed at 5 mm and 10 mm drop are attributable to BMI or the type of footwear used. Previous studies have linked weight gain with balance impairments. Teasdale [39] observed that individuals with obesity improved their stability after losing weight. Corbeil et al. [40] also found that weight gain causes a forward shift in body mass, which forces the ankles to perform torsional movements to maintain balance, thereby increasing the risk of falls. In our sample, the average BMI was 25.3, which falls within the normal range, suggesting that the CoP oscillations in our participants were low.

In line with previous research, mediolateral control emerged as a particularly relevant domain in postural regulation. Variables such as RMS-ML, V-ML, and ellipse areas have been highlighted in the literature as sensitive indicators of balance alterations [41, 42]. Our results support the importance of emphasizing these metrics when assessing the effects of footwear drop, which is consistent with previous findings on the influence of footwear and obesity on postural stability [43, 44]. Furthermore, potential interaction effects were not analyzed in depth in the present study. These interactions could reveal whether the influence of footwear drop varies across sex or according to body composition. Future studies should address these aspects using designs and statistical models that allow for the exploration of such interactions, thereby providing a more comprehensive understanding of inter-individual variability in postural control [45].

## Liminations

First, the cross-sectional design of this study only allowed the assessment of immediate effects of footwear drop, without evaluating potential long-term adaptations. This limits the interpretation of the results, since adaptations to different heel-to-toe drops may occur over time.

Second, the population of this study consisted exclusively of healthy young adults, which limits the generalization of the results. Therefore, the findings cannot be directly extrapolated to other populations, such as older individuals, people with musculoskeletal or neurological pathologies, or high-performance athletes, in whom the response to footwear drop may differ.

Another limitation of this study is that the sample was not homogeneous with respect to BMI or the distribution across drop conditions. This lack of balance may have influenced the variability of the results and reduced the strength of the conclusions. Future studies should consider stratified sampling or larger cohorts that allow for a more homogeneous distribution, in order to better isolate the effects of BMI and footwear drop on postural control.

Finally, it should be acknowledged that this study was conducted in a healthy young population using a cross-sectional design. While this approach provides valuable preliminary insights, it limits the applicability of the findings to clinical or higher-risk populations, such as older adults, individuals with diabetes, or those prone to falls. These groups may exhibit different biomechanical or neuromuscular responses due to age-related or pathological factors. Future research should therefore aim to include longitudinal designs and clinical cohorts, which would not only enhance external validity but also increase the clinical relevance and impact of the results.

## Conclusions

Regarding the quantification of CoP oscillations at the three drop levels studied, it is concluded that CoP oscillations vary depending on the drop used. Greater instability in the ML direction is observed when comparing the 0 mm and 5 mm drop conditions. This finding highlights the importance of evaluating an individual’s balance prior to purchasing sports footwear with a specific drop height, especially in athletes who may have balanced impairments.

Concerning the CoP oscillations based on sex, the results indicate that biological sex influences CoP oscillations, suggesting that the observed differences in balance cannot be solely attributed to the drop. These results show that postural control differs between men and women, which could constitute an area of interest for future research in this field.

Regarding the influence of height on CoP oscillations, the data obtained suggest that stature is a relevant factor in CoP oscillations. Therefore, footwear with a higher drop may affect an individual’s balance, underscoring the importance of considering this variable in sports footwear design.

Finally, when analyzing CoP oscillations in relation to BMI, a direct relationship between an increased BMI and greater static instability was found, as reflected in CoP oscillations. This result underscores the importance of considering BMI in obesity prevention strategies, as individuals with a higher BMI exhibit greater instability compared to those with a normal weight.

## Data Availability

No datasets were generated or analysed during the current study.
